# Magnetic interactions and *in vitro* study of biocompatible hydrocaffeic acid-stabilized Fe–Pt clusters as MRI contrast agents†

**DOI:** 10.1039/c8ra00047f

**Published:** 2018-04-19

**Authors:** N. Kostevšek, S. Hudoklin, M. E. Kreft, I. Serša, A. Sepe, Z. Jagličić, J. Vidmar, J. Ščančar, S. Šturm, S. Kobe, K. Žužek Rožman

**Affiliations:** Department for Nanostructured Materials, Jožef Stefan Institute Jamova 39 Ljubljana Slovenia nina.kostevsek@ijs.si; Institute of Cell Biology, Faculty of Medicine, University of Ljubljana Vrazov trg 2 Ljubljana Slovenia; Department for Condensed Matter Physics, Jožef Stefan Institute Jamova 39 Ljubljana Slovenia; Institute of Mathematics, Physics and Mechanics & Faculty of Engineering and Geodesy, University of Ljubljana Jadranska 19 1000 Ljubljana Slovenia; Department for Environmental Sciences, Jožef Stefan Institute Jamova 39 Ljubljana Slovenia; Jožef Stefan International Postgraduate School Jamova 39 Ljubljana Slovenia

## Abstract

A detailed magnetic study of separated Fe–Pt NPs and Fe–Pt clusters was performed to predict their optimal size and morphology for the maximum saturation magnetization, a factor that is known to influence the performance of a magnetic-resonance-imaging (MRI) contrast agent. Excellent stability and biocompatibility of the nanoparticle suspension was achieved using a novel coating based on hydrocaffeic acid (HCA), which was confirmed with a detailed Fourier-transform infrared spectroscopy (FTIR) study. An *in vitro* study on a human-bladder papillary urothelial neoplasm RT4 cell line confirmed that HCA-Fe–Pt nanoparticles showed no cytotoxicity, even at a very high concentration (550 μg Fe–Pt per mL), with no delayed cytotoxic effect being detected. This indicates that the HCA coating provides excellent biocompatibility of the nanoparticles, which is a prerequisite for the material to be used as a safe contrast agent for MRI. The cellular uptake and internalization mechanism were studied using ICP-MS and TEM analyses. Furthermore, it was shown that even a very low concentration of Fe–Pt nanoparticles (<10 μg mL^−1^) in the cells is enough to decrease the *T*_2_ relaxation times by 70%. In terms of the MRI imaging, this means a large improvement in the contrast, even at a low nanoparticle concentration and an easier visualization of the tissues containing nanoparticles, proving that HCA-coated Fe–Pt nanoparticles have the potential to be used as an efficient and safe MRI contrast agent.

## Abstract 2

Study of magnetic interactions revealed optimal size and morphology of Fe–Pt nanoparticles. Novel biocompatible hydrocaffeic acid coating was used to prepare highly efficient and safe MRI contrast agent, which was proven by *in vitro* study.

## Graphical abstract

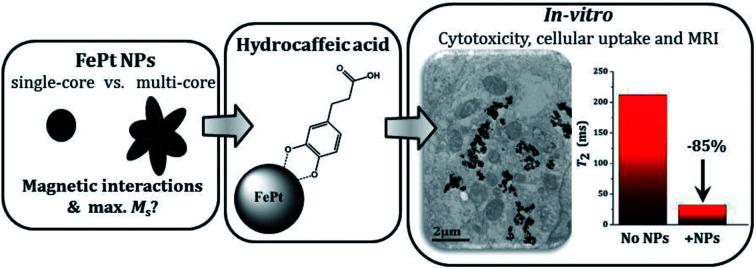

## 1. Introduction

The magnetic properties of the fcc (face-centred cubic) Fe–Pt system include a high Curie temperature and a high bulk saturation magnetization (*i.e.*, about 1140 emu cm^−3^ = 75 emu g^−1^ (*ρ* = 14 kg m^−3^)),^1–3^ which along with its good chemical stability^2,4^ promises the possibility of diverse implementations in biomedicine, *e.g.*, use as a contrast agent in MRI, separation, immunoassays, cell targeting, drug delivery, hyperthermia and magnetic particle imaging.^5^ The value of the material's magnetization has a strong influence on the performance of the nanoparticles (NPs) in all of the above-mentioned applications. The *m*_s_ of Fe–Pt NPs can be increased by increasing their size;^6^ however, in this direction we are limited by the superparamagnetic size limit. This limitation can be bypassed by the formation of controlled clusters of smaller superparamagnetic Fe–Pt NPs.^7^ This approach was successfully applied in our previous study, where the Fe–Pt NPs' magnetization, which equalled 8 emu g^−1^, was increased to 19.5 emu g^−1^ for Fe–Pt clusters by changing the ratio and volume of the added surfactants (oleic acid and oleylamine), while keeping the Fe–Pt NPs in the superparamagnetic regime. In this study we have investigated the mechanism behind the increase in the *m*_s_ in the case of Fe–Pt clusters and thereby tried to answer the question “what is the maximum *m*_s_ that can be achieved in the superparamagnetic regime for Fe–Pt NPs”. To do this, the magnetic interactions between the Fe–Pt NPs were evaluated using temperature-dependent field-cooled and zero-field-cooled (FC/ZFC) magnetic susceptibility curves.

There are only a limited number of studies that can be found in the literature referring to testing the cytotoxicity of Fe–Pt NPs.^8–17^ In all these examples only three different coatings were used, mostly cysteamine, followed by cysteine and three examples of PEG-based coatings. This demonstrates that Fe–Pt NPs are promising candidates for several biomedical applications, but there is a clear gap in the development of new biocompatible NPs coatings, which would shift the concentration range where NPs show no cytotoxic effect to the highest possible values. Literature reports on Fe oxide NPs have shown that the dopamine provides a strong coordination to the NP surface,^18,19^ while the carboxylic group is responsible for the high water solubility and suspension stability across the wide pH range.^18^ We have demonstrated in the previous work on the example of the zwitterionic dopamine sulphonate (ZDS) ligand that dopamine-based ligands are good candidates for the functionalization and stabilization of Fe–Pt NPs.^6^ As the continuation of the search for a simple-to-synthesize, efficient and biocompatible ligand, we propose in this study a new ligand from the family of dopamine-based ligands, *i.e.*, hydrocaffeic acid (HCA). HCA was selected as a ligand because of its natural origin. Polyphenol-rich foods, such as vegetables and fruits, contain HCA.^20,21^ Moreover, HCA is a metabolite of caffeic acid and has potent antioxidant properties, even more effective than the caffeic acid. To the best of our knowledge, this study is a first report about functionalization of Fe–Pt NPs with HCA ligand.

A change in the environment can drastically influence the NPs' stability and, consequently, their performance. Therefore, in order to better understand the behaviour of the NPs in the cellular environment and their influence on the cell viability, an *in vitro* study on the cancer urothelial RT4 cell line was performed. First, possible cytotoxicity of HCA-functionalized Fe–Pt NPs was evaluated over a broad concentration range (10–550 μg mL^−1^ of Fe–Pt NPs). Secondly, the cell viability was followed up to one week after the NPs' removal to asses any possible delayed cytotoxic effect. Then, the process of the NPs' internalization and the mechanism of the cellular uptake was determined. Finally, to test the performance of the Fe–Pt NPs as the contrast agents, *in vitro* MRI measurements were performed and the effect of the NPs' presence in the cells on the shortening of the relaxation times was evaluated.

Briefly, this work represents the entire workflow, from the optimization of the magnetic performance by forming clusters of superparamagnetic NPs, an investigation of the magnetic interactions responsible for the increase in the *m*_s_, *via* the successful functionalization of NPs with biocompatible HCA ligand, which was supported with a full characterization, to an evaluation of the possible cytotoxicity and a demonstration of their potential for use as a safe contrast agent in MRI.

## 2. Experimental

### 2.1. Materials for the preparation of HCA-Fe-Pt NPs

For the synthesis of the Fe–Pt NPs, platinum acetylacetonate Pt(acac)_2_ (Merck), iron acetylacetonate Fe(acac)_3_ (>99.9% Sigma-Aldrich), benzyl ether (>98%, Merck), oleic acid (OA, >99%, Sigma-Aldrich), oleylamine (OLA, 70%, Sigma-Aldrich), 1,2-hexadecanediol (90%, Sigma-Aldrich), hexane (>95%, Sigma Aldrich) and ethanol absolute anhydrous (>99.9%, Carlo Erba Reagents) were used. For the ligand-exchange reaction and the functionalization of the Fe–Pt surface with the HCA ligand the following reagents were used: tetrahydrofuran (THF, anhydrous, >99.9%, Sigma Aldrich), hydrocaffeic acid (HCA, 3-(3,4-dihydroxyphenyl)propionic acid, >98%, Sigma Aldrich) and NaOH (anhydrous, >98%, Sigma Aldrich).

#### Synthesis of Fe–Pt NPs

The Fe–Pt NPs were prepared as described in our previous article.^6^ Briefly, at room temperature, 0.5 mM of Pt(acac)_2_ and 1 mM of Fe(acac)_3_ were added into a round-bottom flask containing 20 mL of benzyl ether. Then 4 mL of oleic acid and 4 mL of oleylamine were added. Before heating to 260 °C for 30 min the reducing agent 1,2-hexadecanediol (2.3 mmol) was added at 160 °C. The black product was precipitated by adding absolute ethanol and separated by centrifugation (6000 rpm/10 min) and then re-dispersed in hexane. For the preparation of Fe–Pt clusters the oleic-acid-to-oleylamine ratio was changed, *i.e.*, 2 mL of oleic acid and 4 mL of oleylamine, while all the other parameters were kept the same as in the synthesis of separated Fe–Pt NPs.

#### Ligand-exchange reaction

The as-prepared hydrophobic Fe–Pt NPs (20 mg) were dispersed in 1 mL of THF. The solution of ligand was prepared by dissolving 50 mg of HCA in 5 mL of THF. Hydrophobic NPs were added dropwise to the solution of the ligand and the reaction mixture was then stirred at 50 °C for 3 h to complete the reaction. Upon cooling the reaction mixture to room temperature, 0.5 mL of 0.5 M NaOH was added to precipitate the NPs, which were collected by centrifugation and re-dispersed in water.

### 2.2. Methods

For the synthesis of the Fe–Pt NPs, a hemispherical heating mantle (model WiseTherm WHM 12112 from Witeg Labortechnik GmbH) connected to a temperature controller (J-KEM, model 310) with a J-type Teflon thermocouple was used. The samples were characterized using a (scanning) transmission electron microscope (TEM Jeol JEM-2010F) equipped with energy-dispersive X-ray spectroscopy (EDXS). Low-temperature magnetic measurements were performed on a Quantum Design MPMS-XL-5 SQUID magnetometer. With the thermogravimetric analyses (TG analyser NETZSCH STA 449 C/6/G Jupiter) the amount of organic matter in the sample was determined to be 30%. The magnetization values are reported for the mass of Fe–Pt in the sample after the subtraction of the organic content. Fourier-transform infrared spectroscopy (FTIR) measurements were performed using a Spectrum 400 spectrometer (Perkin Elmer, USA). The spectra were recorded on dried samples in the wavenumber range 4000–650 cm^−1^.

#### ICP-MS analysis

The total concentrations of Fe and Pt in the analysed samples were determined by mass spectrometry with inductively coupled plasma (ICP-MS, Agilent 7700 ICP-MS instrument, Agilent Technologies, Tokyo, Japan). Stock solutions of Pt (1000 μg Pt per mL in 8% HCl) and Fe (1000 μg Fe per mL in 2–3% HNO_3_), all obtained from Merck (Darmstadt, Germany), were diluted with water for the preparation of fresh calibration standard solutions. For the determination of Fe and Pt in an aqueous suspension of Fe–Pt NPs, to a 0.5 mL of NPs suspension 1.5 mL of hydrochloric acid (30% HCl, suprapure) and 0.5 mL of nitric acid (65% HNO_3_, suprapure) were added. The sample was heated on a hotplate (C-MAG HP10, IKA, Germany) at 100 °C for 10 min until the colour of the solution turned yellow. After the digestion, the sample was filled with MilliQ water to a final volume of 10 mL and appropriately diluted prior to the ICP-MS measurements. Digestion was performed in duplicate. For the determination of the Fe and Pt concentrations in the cells' suspensions, 2 mL of sample was digested with 0.75 mL of HCl, 0.5 mL of HNO_3_ and 1.25 mL of hydrogen peroxide (30% H_2_O_2_, suprapure) and heated at 90 °C overnight in an oven (Binder GmbH, Tuttlingen, Germany). After the digestion, the sample was filled up with MilliQ water to a final volume of 10 mL and appropriately diluted prior to the ICP-MS measurements. All the dilutions of the samples were made with ultrapure water (18.2 MΩ cm) obtained from a Direct-Q 5 Ultrapure water system (Merck Millipore, Milford, MA, USA). Nitric acid (65% HNO_3_), hydrochloric acid (30% HCl) and hydrogen peroxide (30% H_2_O_2_) were obtained from Merck Millipore, Milford, MA, USA.

### 2.3. *In vitro* experiments, cell viability and TEM

Cell line of human cancer urothelial cells, derived from papillary neoplasm, was established as described previously.^22^ Human-bladder papillary urothelial neoplasm (RT4) cells were seeded with a density of 5 × 10^4^ cells per cm^2^ (TPP, Trasadingen, Switzerland) and grown in A-DMEM/F12 (1 : 1) (Gibco), 5% fetal bovine serum (FBS; Gibco) (FBS), 4 mM GlutaMAX (Gibco), 100 U mL^−1^ penicillin, and 100 μg mL^−1^ streptomycin and were maintained at 37 °C in a humidified 5% CO_2_ atmosphere for 1 week before the experiments. All the HCA-Fe–Pt NP incubation experiments were performed in the same culture medium as used to establish the urothelial RT4 cell model.

#### Cell viability

A Trypan-blue viability assay was used to determine the viability of the RT4 cells after HCA-Fe–Pt NP exposure. The cells were grown in 6-well plates and then incubated with different concentrations of NPs (10, 50, 100, 250 and 550 μg Fe–Pt per mL) for 24 h. After the incubation the cells were washed to remove the non-internalized NPs, trypsinized until all the cells were detached and following the manufacturer's instructions immediately stained with Trypan-blue dye, which labels only dead cells. The live and dead cells were than counted manually under an inverted light microscope (Leica). The percentage of viable cells (% viability) in a given sample was determined as the ratio between the number of viable cells and all the cells in the sample. In addition, cell viabilities were also calculated 7 days after the incubation with the NPs. The cell viabilities were analyzed in two independent experiments, each with at least four technical repeats.

#### Transmission electron microscopy

The RT4 cells were incubated with 100 μg mL^−1^ HCA-Fe–Pt NPs for 1 or 12 h at 37 °C, washed to remove the non-internalized NPs and then fixed or cultured for an additional 24 h. The samples were fixed with a mixture of 3% paraformaldehyde and 3% glutaraldehyde in 0.1 M cacodylate buffer overnight at 4 °C and subsequently in 2% osmium tetraoxide for 1 h at room temperature. The samples were stained en bloc with 2% uranyl acetate for 1 h, dehydrated in a graded series of ethanol solutions and embedded in Epon 812 resin (Serva Electrophoresis, Heidelberg, Germany). Semi-thin (1 μm) and ultra-thin (65 nm) sections were cut using an Ultracut UCT microtome (Leica, Austria). The semi-thin sections were contrasted with toluidine blue and examined under a light microscope to determine the positions of the cells. Ultra-thin sections were examined with a Philips CM100 TEM.

#### *In vitro* MRI

Relaxation-time measurements were made on an NMR/MRI system consisting of a 9.4-T superconducting magnet (Jastec, Kobe, Japan) and a Redstone NMR spectrometer (Tecmag, Houston TX, USA). The *T*_1_ relaxation times were measured using an inversion-recovery sequence with 16 different inversion times, ranging from 100 μs to 10 s, while the *T*_2_ relaxation times were measured using the Carr Purcell Meiboom Gill (CPMG) sequence with multiple spin-echoes. The *T*_1_ and *T*_2_ relaxation times were calculated from the best fits between the measurements and the corresponding model for either *T*_2_ relaxation (exponential dependency of the echo-signal on the echo number) or *T*_1_ relaxation (dependency of the inversion recovery signal on the inversion time). The calculations were performed using the Origin program (OriginLab Corporation, Northampton MA, USA). The cells were incubated with different concentrations of HCA-Fe–Pt NPs (0, 10, 50 and 100 μg mL^−1^) for 12 h. After the incubation the cell-culture media containing the NPs was removed, the cells were washed to remove all NPs that were not taken up and then densified into a pellet with centrifugation. Suspensions of the cells were centrifuged in tubes for the relaxivity measurements (height 70 mm and diameter 8 mm) at 1300 rpm for 5 minutes. The supernatant was removed and an as-prepared pellet of cells was used in the measurements.

## 3. Results and discussion

### 3.1. Synthesis and magnetic characterization

**Fig. 1:**
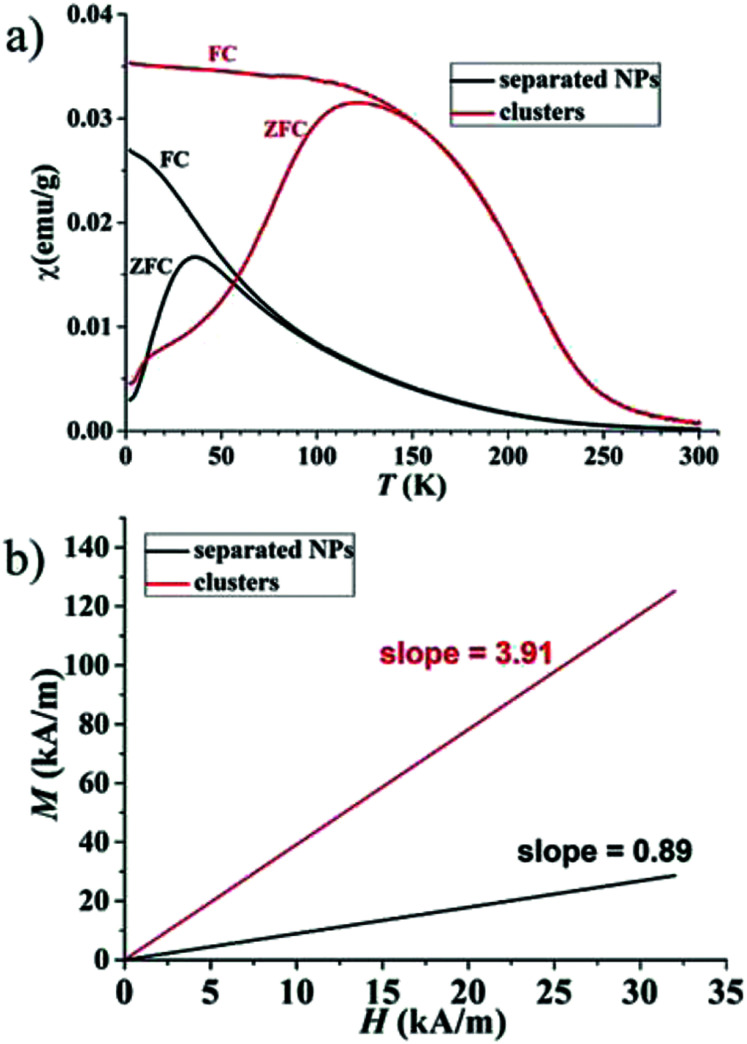
(a) Temperature-dependent field-cooled and zero-field-cooled (FC/ZFC) magnetic susceptibility curves for separated Fe–Pt NPs and Fe–Pt clusters and (b) initial magnetization at low *H* (0–32 kA m^−1^) measured at 300 K.

Separated Fe–Pt NPs and Fe–Pt clusters were prepared according to the optimized procedure described in the literature^6^ by changing the ratio between the two surfactants, oleic acid (OA) and oleylamine (OLA), that are used in the synthesis. TEM images of the separated Fe–Pt NPs and Fe–Pt clusters are shown in Fig. S-1a and S-1b in the ESI,† respectively. The structure of the clusters was proven using high-resolution TEM analysis in our previous study.^6^ The average size (defined as the average diameter) of the separated Fe–Pt NPs measured on the basis of 30 individual particles was found to be 7 ± 1 nm. In the case of the Fe–Pt clusters the average size was estimated by measuring the average diameter of the body, and the average particle size was determined to be 20 ± 3 nm. The elemental composition determined from the obtained EDXS data was Fe_25±1_Pt_75±1_ and Fe_30±1_Pt_70±1_ for the separated Fe–Pt NPs and Fe–Pt clusters, respectively. A slightly higher content of Fe in the case of the Fe–Pt clusters can be attributed to the lower content of OA during the synthesis, since it was suggested that OA is a coordinative ligand for the Fe atoms, and OLA is said to coordinate the Pt intermediates.^23^ Therefore, a smaller amount of OA would result in fewer coordinated Fe metal intermediates in the reaction mixture and hence NPs with a slightly higher Fe content. The step from smaller separated Fe–Pt NPs to the larger, Fe–Pt clusters (>20 nm) leads to an increase in the magnetization *m*_(1.5T)_ from 8 to 19.5 emu g^−1^ (Fig. S-1c†), without exceeding the superparamagnetic limit. In order to gain an insight into the magnetic interactions acting between the Fe–Pt NPs, which can be used to explain the mechanism behind the increase in the *m*_s_ in the case of Fe–Pt clusters, the temperature-dependent field-cooled and zero-field-cooled (FC/ZFC) magnetic susceptibility curves were recorded and are shown in Fig. 1a. The blocking temperatures (*T*_b_) for the separated Fe–Pt NPs and Fe–Pt clusters were determined to be 36 K and 121 K, respectively. This is in good agreement with the Néel relaxation theory, where larger particles have a higher *T*_b_. The broader peak in the case of the Fe–Pt clusters indicates a broader size distribution, which is consistent with the results obtained from the TEM analysis. Moreover, *T*_b_ can be shifted towards higher temperature due to the presence of the magnetic dipole interactions.

The spin structure of the NPs can be affected by the inter-particle interactions, which are mainly long-range dipole–dipole interactions and the exchange interactions through the surface of the particle. If the magnetic susceptibility (FC curve) becomes temperature independent at *T* ≪ *T*_b_ and reaches saturation, this indicates the presence of inter-particle dipole interactions. It is clear from the graph in Fig. 1a that for both samples the susceptibility of the FC curves in the range *T* ≪ *T*_b_ increases with the decreasing temperature. Even though complete saturation is not reached, it can be inferred from the FC curves that the Fe–Pt NPs in dry form are weakly interacting. The Fe–Pt clusters show a less steep curve in that temperature range, which can be attributed to the stronger dipole interactions, as in the case of the separated Fe–Pt NPs. The larger dipole interactions in the case of the larger NPs might arise due to the higher saturation magnetization.^24^ Similar behaviour was shown for the case of the 3- and 9 nm-sized fcc Fe–Pt NPs, where both samples have non-negligible dipole interactions, the effect being smaller in the smaller NPs.^24^ It must be pointed out that the samples for the ZFC/FC measurements were in a dry form with the inter-particle distance being reduced; therefore, significant dipole interactions at lower temperature could be expected. If, however, the magnetic dipolar interactions would also be significant at room temperature, they would compromise the superparamagnetic properties and the colloidal stability of the suspension, which was not the case in our study. We additionally presume that in the case of separated Fe–Pt NPs in the suspensions the direct exchange interactions between the individual NPs might be neglected due to the surfactant coating, which prevents their direct contact.^25^ However, in clusters the exchange interactions, *i.e.*, the intra-particle collective behaviour, can be present, which leads to an enhancement in the effective magnetic moment of the clusters, as was already observed by Lartigue *et al.*^26^ in Fe-oxide clusters. The clustering of the NPs would have an influence on the effective magnetic moment of the NP (*μ*_eff_), which can be determined using the following procedure. From the magnetic hysteresis measurements (Fig. S-1c†), the initial susceptibility of the samples (*χ*_0_) can be calculated using the slope of the *M* (kA m^−1^) *vs. H* (kA m^−1^) curve at low *H*. The mass magnetization (emu g^−1^) was converted to volumetric magnetization (emu cm^−3^) by assuming a density of Fe–Pt NPs equal to 14 g cm^−3^. For the calculation purposes in this case *M* (emu cm^−3^) and *H* (*T*) were converted into SI units (kA m^−1^). *M*–*H* curve at low *H* (0–32 kA m^−1^) for the separated Fe–Pt NPs and Fe–Pt clusters is shown in Fig. 1b. The initial susceptibility (*χ*_0_) was found to be 0.89 and 3.93 for the separated Fe–Pt NPs and Fe–Pt clusters, respectively. Furthermore, by using the relation1
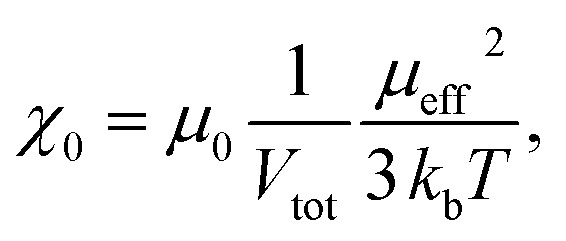
the effective magnetic moment for the nanostructure (*μ*_eff_, can be calculated, where *V*_tot_ is the total volume of a NP, *k*_b_ is the Boltzmann constant (1.38 × 10^−23^ J K^−1^), *T* is the temperature (K) and *μ*_0_ is the permeability of a vacuum (4π × 10^−7^ N A^−2^)).^27,28^ Reorganization of eqn (1) gives an expression for the direct calculation of *μ*_eff_:2
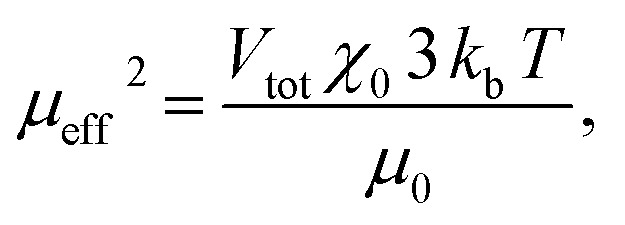


The effective magnetic moment of the NPs (*μ*_eff_) was then calculated by using eqn (2). The volume of the Fe–Pt NPs was estimated to be *V*_(separated)_ and *V*_(clusters)_ 1.13 × 10^−25^ m^3^ and 1.76 × 10^−24^ m^3^, respectively. The calculated effective magnetic moments are *μ*_eff-(separated)_ = 3 × 10^−20^ A m^2^ and *μ*_eff-(clusters)_ = 26 × 10^−20^A m^2^. From the above calculations it can be seen that the Fe–Pt clusters possess a higher effective magnetic moment than the separated ones. Moreover, if the particles in the clusters structure are assumed to have the same magnetic moment (magnetic moment of the particle, *μ*_p_) and are magnetically non-interacting, we would expect that the effective magnetic moment of the cluster (*μ*_eff_) would follow the statistical relation for randomly oriented independent (non-interacting) particle magnetic moments (*μ*_p_) according to the relation:^27^3
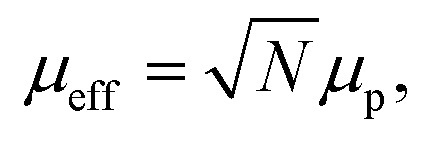
where *N* is the number of particles in the cluster. If we assume that the average number of particles in the cluster is 8, the *μ*_eff-(cluster)_ = 2.8*μ*_eff-(separated)_, which is less than was shown experimentally (*μ*_eff-(cluster)_ ≈ 8*μ*_eff-(separated)_). This can be attributed to the collective magnetic behaviour of the Fe–Pt particles^26^ and/or to the slight increase in the particle size, which was confirmed by TEM.

**Fig. 2:**
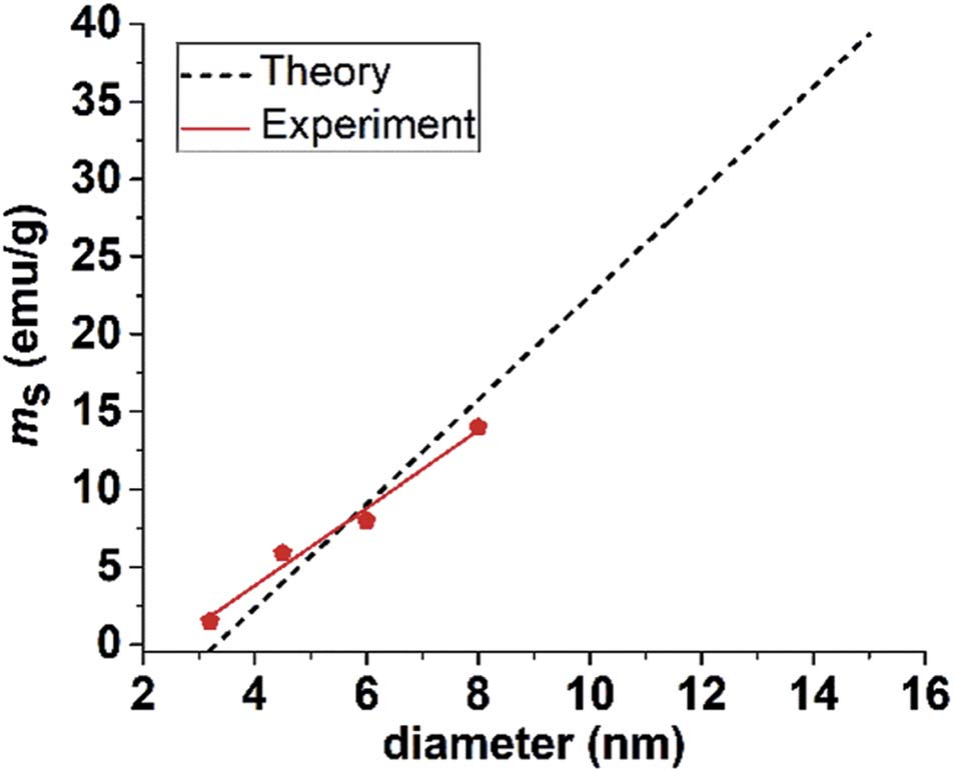
Graph indicating theoretically calculated and experimentally obtained saturation magnetization for separated Fe–Pt NPs with different diameters.

Furthermore, surface effects can also significantly influence the magnetic properties of the NPs. In the case of clusters, the surface-to-volume ratio is reduced due to coalescence of the individual particles, which could also contribute to the increase of the magnetization. According to eqn (4) the saturation magnetization of NPs (*m*_s_) is proportional to the NPs' size:

4 *m_s_* = *M_s_* [(*r − d*)/*r*]^3^,

where *r* is the radius of the NP and *d* is the thickness of the disordered surface spin layer. Eqn (4) shows that the maximum magnetization can be obtained when the disordered surface spin layer disappears. The theoretically calculated *m*_s_ for separated Fe–Pt NPs obtained from eqn (4) are shown in Fig. 2 (black line), where *r* = 1.6–7.5 nm, *d* = 1.5 nm and *m*_s_ = 75 emu g^−1^ were used. The experimental *m*_s_ values (*m*_s_ = 1.5–14 emu g^−1^) for the separated Fe–Pt NPs in the range *r* = 1.6–4 nm were taken from our previous study^6^ and are plotted in a graph shown in Fig. 2 (red line). Combining all these data revealed a good match between theory and experiment. The Fe–Pt clusters have *m*_s_ = 19.5 emu g^−1^. Plotting this value on the graph in Fig. 2, a theoretical NP with a diameter of 9.7 nm is obtained. This indicates that the cluster magnetically behaves as a separated particle with a size of 9.7 nm. The TEM analysis of the Fe–Pt clusters showed that the size of the individual particles is in the range 8 ± 1 nm, which is “slightly” less than it was calculated using the above equation. From this it could be assumed that the magnetic ordering due to the presence of the intra-particle exchange interactions increases the magnetic diameter of the particles. Therefore, we can conclude that the observed increment in the magnetization of the clusters has a basis in the reduction of their surface disorder (smaller surface-to-volume ratio) and/or in the magnetic exchange interactions between the individual particles.

As no reports about the superparamagnetic size limit (*D*_SPL_) for fcc Fe–Pt can be found in the literature, we used an experimental *T*_b_ to calculate *D*_SPL_, which was found to be approximately 10 nm. Details can be found in the ESI.† Based on the presented results we can conclude that the highest possible magnetization for our Fe–Pt system would be theoretically obtained by fabricating separated Fe–Pt NPs with a particle size just below *D*_SPL_ and combine them together into clusters, introducing the collective magnetic behaviour without crossing the SPL. For example, the calculated *m*_s_ for separated NPs with a size around SPL (10 nm) is 23 emu g^−1^. Therefore, due to the presence of the exchange interactions between the individual cores, combining 10 nm-sized particles in clusters would lead to an even further increase in *m*_s_. However, this value is expected to still be much lower than the bulk saturation magnetization (75 emu g^−1^) that cannot be obtained in the superparamagnetic regime due to the prevalent surface effects. An experimental hint about the maximum size of the Fe–Pt clusters can be seen from the work of Green *et al.*,^7^ where Fe–Pt clusters with a size above approximately 40 nm and *m*_s_ = 29 emu g^−1^ already showed evidence of ferromagnetism at room temperature.

### 3.2. Surface functionalization with HCA

**Fig. 3:**
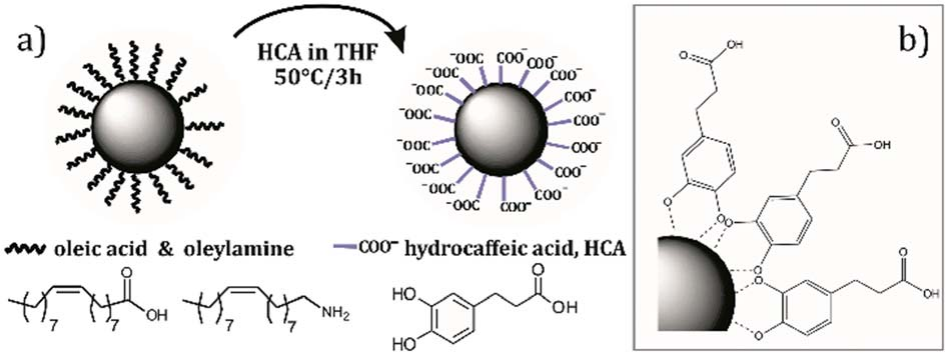
(a) Schematic representation of a ligand-exchange reaction with HCA in THF and (b) chelate bonding of a HCA on Fe–Pt NPs.

The as-synthesized hydrophobic Fe–Pt NPs were transferred to an aqueous phase by using the HCA ligand dissolved in tetrahydrofuran (THF), as schematically shown in Fig. 3a. Fig. S-2 in the ESI† evidently shows that after the ligand-exchange reaction the separated Fe–Pt NPs are negatively charged (from −10 mV at pH = 2 and −50 mV at pH = 12) across the whole pH range that was used in the measurements. The negative zeta-potential is related to the deprotonation of the carboxylic group of the HCA ligand. High measured values of the zeta-potential enable a strong electrostatic repulsion between the NPs, which results in a persistent suspension stability for a period of several months. Higher relative zeta-potential values in the case of Fe–Pt clusters (from −38 mV at pH = 2 to −70 mV at pH = 12) can be attributed to a larger size of NPs where a larger surface allows for the attachment of more HCA molecules and, consequently, a higher surface charge can be obtained.

**Fig. 4:**
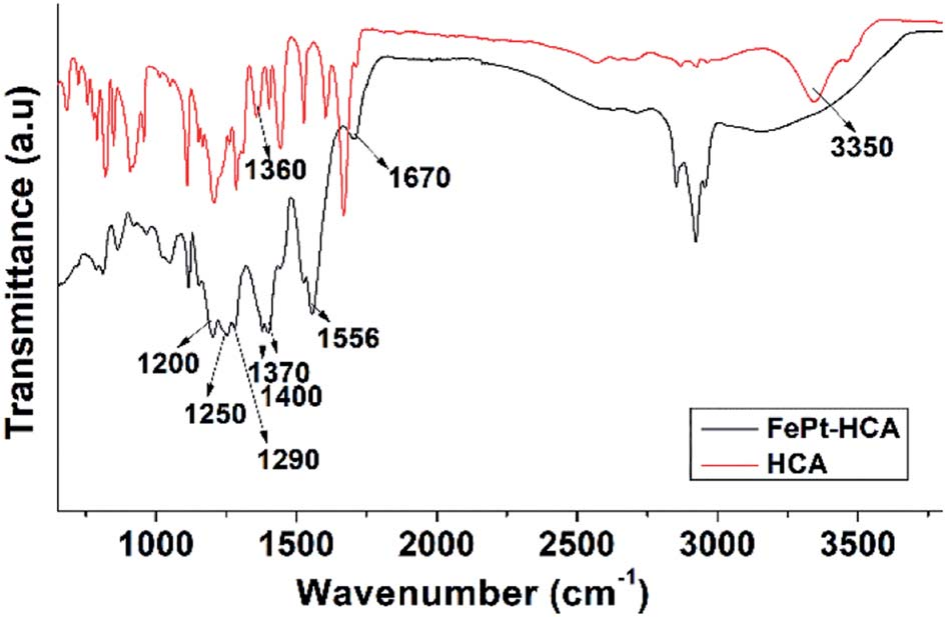
FTIR spectra of pure HCA and HCA-functionalized Fe–Pt NPs.

In order to examine the bonding between the different ligands (OLA, OA and HCA) and the NP's surface a detailed FTIR study was performed. Before the ligand-exchange reaction OLA and OA ligands can be found on the Fe–Pt NP surfaces. Individual FTIR spectra of the pure ligands, *i.e.*, OA and OLA, with identified peaks are shown in Fig. S-3 and S-4 in the ESI,† respectively. A detailed FTIR study of the OA and OLA bonding on the surface of Fe–Pt NPs can be found in the work of Shukla *et al.*^29^ FTIR spectra with a detailed description of assigned peaks for the as-synthesized OA- and OLA-stabilized Fe–Pt NPs together with the FTIR spectra of pure OA and OLA are shown in Fig. S-5 in the ESI.† A ligand-exchange reaction with HCA was performed to transfer the Fe–Pt NPs into the water phase. The FTIR spectrum of the pure HCA ligand was recorded (Fig. S-6 in the ESI†) and a detailed explanation of the assigned peaks is given in the ESI.† From Fig. 4 it can be seen that the FTIR spectrum of the HCA-functionalized Fe–Pt NPs differs significantly from the FTIR spectra of the pure HCA ligand. The HCA ligand has two possible bonding sites with the NP's surface: the COOH group and the OH catechol groups; therefore, the major difference in the peak positions are expected to be related to the peaks correlated to those functional groups.

The first distinct change in the spectrum of the HCA-functionalized Fe–Pt NPs occurs due the deprotonation of the carboxylic group. More precisely, peaks correlated to the *ν*(C=O) = 1670 cm^−1^ and *ν*(C–O) = 1290 cm^−1^ stretch mode of the protonated carboxylic group diminish and new peaks for the symmetric stretching at *ν*_asym_(COO–) = 1556 cm^−1^ and asymmetric stretching of the carboxylate at *ν*_sym_(COO–) = 1370 and 1400 cm^−1^ appear.^30^ Splitting of the peaks often resulted from the formation of different carboxylate-metal inner-sphere complexes.^31^ The separation in wavenumber Δ*ν*_(aysm–sym)_ for the asymmetric and symmetric stretching of carboxylate bands can be used to elucidate the bonding coordination. In general, Δ*ν* values greater than about 200 cm^−1^ are thought to indicate monodentate binding, whereas 180–150 cm^−1^ indicates binuclear bidentate (bridging) complexes, and <100 cm^−1^ indicates mononuclear bidentate binding (chelation).^30^ The separation in wavenumber Δ*ν*_(aysm–sym)_ was determined to be 186 and 156 cm^−1^ for the first and second symmetric peaks, respectively. Therefore, it can be assumed that HCA ligand bonds to the NP's surface in the bidentate (bridging) form. The peak splitting might be attributed to the formation of HCA-Fe and HCA-Pt complexes at the Fe–Pt NP's surface. The presence of the peak that corresponds to the stretching mode (*ν*(C–OH) = 1200 cm^−1^) indicates that the aryl oxygen stretching is not affected by the catechol adsorption on the NP's surface. Moreover, the peak at (*ν*(CO–M) = 1250 cm^−1^) occurs when the catechol binds covalently to the metal as a catechol anion. In the case of the HCA-functionalized Fe–Pt NPs the disappearance of both peaks, the stretching vibration of the OH groups (broad peak at 3200–3500 cm^−1^) and the bending vibrations of the catechol OH groups (*ν*(C–OH) = 1360 cm^−1^) that are present in pure HCA, indicates bonding of the HCA to the Fe–Pt NPs surface with the catechol OH groups.^32^ From the obtained spectroscopic data it can be concluded that two types of binding structures for the HCA on the Fe–Pt NP's surface are possible, *i.e.*, *via* carboxylic groups or catechol OH groups, which is in accordance with the literature data.^33^ The deprotonation of HCA is an indication of the bonding *via* the carboxylic groups. However, carboxylic groups on the outer surface do not bind to the Fe–Pt NP's surface, providing good suspension stability and a high negative zeta-potential.^33^ It is believed that the majority of the HCA molecules bind to the NP's surface *via* catechol groups,^33^ as schematically shown in Fig. 3b.

### 3.3. *In vitro* studies

#### 3.3.1 Cytotoxicity of the HCA-Fe-Pt NPs

Due to their superior magnetic properties, Fe–Pt clusters were selected to be used in the *in vitro* studies and are subsequently referred to as HCA-Fe–Pt NPs. The cytotoxicity of the HCA-Fe–Pt NPs was evaluated by incubating cells with different concentrations of Fe–Pt NPs (10, 50, 100, 250 and 550 μg Fe–Pt per mL) for 24 h. The cell viabilities obtained after the incubation with NPs for all the tested concentrations are summarized in Table 1.

**Table 1:** Cell viabilities for the RT4 cells after the incubation with HCA-Fe-Pt NPs with different concentrations for 24 h (*n* = 3)

Concentration (μg Fe–Pt per mL)	0	10	25	50	250	550
Cell viability (%)	94.4	94.9	97.1	95.6	96.5	94.8
Std dev.	±3.4	±2.3	±1.5	±0.5	±1.1	±2.1

Even at the highest tested concentration (550 μg Fe–Pt per mL), which is much above the concentration level that is usually used in *in vitro* studies, the cell viability remained high (94.8 ± 2.1%). Comparing these results with the literature data presented in the introduction part it can be seen that the HCA-coated Fe–Pt NPs showed no significant cytotoxicity, even at much higher concentrations than the NPs coated with cysteine^34^ (100 μg Fe–Pt per mL) or cysteamine^11^ (30 μg Fe–Pt per mL), which are the two most commonly used molecules from the studies found in the literature. Because the presence of the NPs may induce a delayed cytotoxic effect, it is important to follow the cell viability of the cells for an extended period of time. Measurements revealed that 1 day and 7 days after the NPs' removal the cell viabilities remained high (>95%, data not shown). From this it can be concluded that due to the presence of the HCA-Fe–Pt NPs no delayed cytotoxic effect was observed.

#### 3.3.2 Time-dependent cellular uptake: microscopy and ICP-MS analysis

**Fig. 5:**
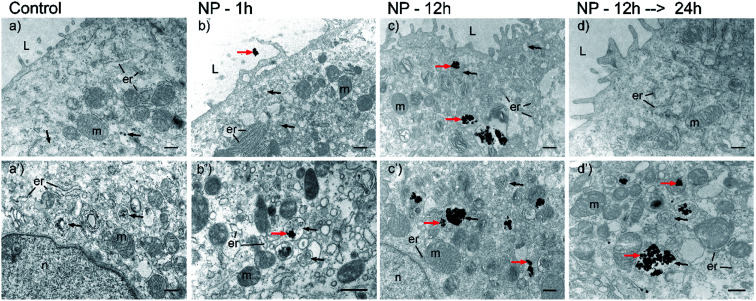
Transmission electron microscopy images of RT4 cells during incubation with HCA-Fe-Pt NPs. Panels a-d show the cell surface and apical region of the cells, while a′–d′ show the central cytoplasm. (a and a′) Control cells before incubation, (b and b′) cells after 1 h of incubation, (c and c′) cells after 12 h of incubation and (d and d′) after the 12 h period of incubation with NPs, followed by 24 h incubation without NPs. Legend: red arrows – HCA-Fe-Pt NPs, black arrows – endosomal compartments, er – endoplasmic reticulum, L – lumen, m – mitochondria, n – nucleus. Bar: 500 nm.

In order to follow the process of the NPs' internalization and to explain the mechanism of the cellular uptake, RT4 cells were incubated with 100 μg mL^−1^ HCA-Fe–Pt NPs for 1 or 12 h, washed to remove the non-internalized NPs and then fixed or cultured for an additional 24 h. After each time point, the samples were analyzed with TEM to visualize the NPs' location in the cells (Fig. 5). TEM images of the control cells are shown in Fig. 5a. TEM images of the cells incubated with HCA-Fe–Pt NPs for 1 h, 12 h are shown in Fig. 5b and c, respectively, and 24 h after 12 h incubation with HCA-Fe–Pt NPs are shown in Fig. 5d. After 1 h of the incubation only a few NPs can be found on the cell membrane (plasma membrane) or internalized (Fig. 5b), while after 12 h the amount of internalized NPs is significantly higher (Fig. 5c). These results revealed a strong time-dependent cellular uptake. The amount of NPs was not significantly changed 24 hours after the 12 h-incubation (Fig. 5d). The NPs were generally no longer on the cell surface; however, they were clearly visible intracellularly in endosomal compartments (Fig. 5d'). Importantly, the cell organelles were, at all time points, well preserved and of normal morphology. Moreover, the NPs were not caught in the intracellular space of the RT4 cell model.

**Fig. 6:**
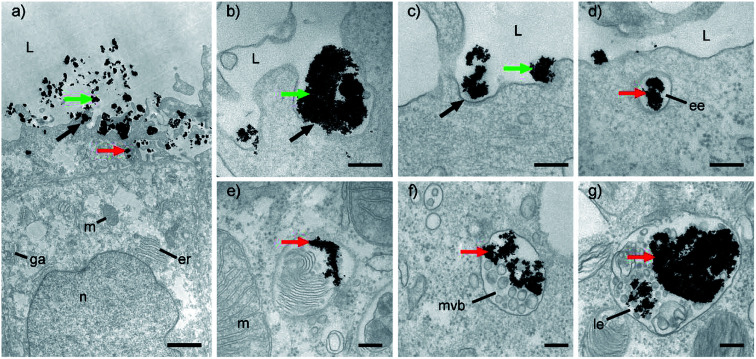
Internalization pathway of HCA-Fe–Pt NPs into the RT4 cells. (a) RT4 cell exposed to NPs. NPs were detected in the lumen (green arrows) and associated with the cell apical plasma membrane, where they formed bigger (b) and smaller (c and d) clusters of NPs. Apical plasma membrane around the NP started to evaginate and then invaginate into the cell's cytoplasm (black arrows), presumably by the mechanism of (b) macropinocytosis and (c) clathrin-dependent endocytotis. (d) Beneath the apical plasma membrane, internalized NPs were seen in early endosomes (ee), which progressively matured into the late endosomes (le; e–g). During endosome maturation, NPs in them accumulated and were mixed with the other cell membranes and components, determined for degradation (e–g). Legend: green arrows – HCA-Fe–Pt NPs in the lumen, red arrows – endocytosed HCA-Fe–Pt NPs, black arrows – invagination of the apical plasma membrane, ee – early endosome, er – endoplasmic reticulum, ga – Golgi apparatus, le – late endosome, m – mitochondria, mvb – multivesicular body, n – nucleus. Bars: (a) 2 μm, (b–g) 200 nm.

Further TEM analyses enabled us to follow the HCA-Fe–Pt NPs internalization pathway, from the cell surface (Fig. 6a–c) into the cells (Fig. 6d–g). NPs were observed in different endosomal compartments, *e.g.*, based on their ultrastructural characteristics, mainly in early and late endosomes (Fig. 6d–g). Regarding the ultrastructural changes of the apical surface, *i.e.*, prominent ruffling of the plasma membrane and engulfment of small NP aggregates, the most likely endocytotic mechanism would be through macropinocytotis, which enables the nonspecific internalization of larger volumes of a fluid phase with intensive plasma membrane evaginations in the form of blebs, ruffles and lamellar evaginations, also in non-phagocytic cells.^35^ Moreover, we have noticed some clathrin-dependent endocytosis (Fig. 6c). Since it is known that NP internalization depends not only on the physicochemical properties of NPs,^36^ the extracellular environment (*e.g.*, pH, ion and protein composition^37^), but also on the cell type^38^ and their differentiation stage,^39,40^ we must also be aware that the utilized endocytotic pathway for HCA-Fe–Pt NPs could be different in other cell types.

Furthermore, in order to quantitatively assess the NPs' uptake, an ICP-MS analysis was made and the concentration of Fe and Pt in the cells was determined. Cells incubated for 12 h with a NP concentration of 100 μg m^−1^ were used in these experiments. Because the cells already naturally contain Fe, samples that were not exposed to the NPs and the cell culture medium were measured as the control samples. No Pt was expected to be present in the control samples. Furthermore, to follow the fate of the NPs for an extended period of time, 1 day and 7 days after the removal of the NPs, an ICP-MS analysis was made and the calculated concentrations of Fe and Pt in the cells are shown in Table 2.

**Table 2:** Fe and Pt concentrations determined with ICP analysis on the samples 0 h, 1 day and 7 days after the NPs' removal (incubation time was 12 h with concentration of HCA-Fe–Pt NPs 100 μg mL^−1^)

	Fe μg per mL	Pt μg per mL
Cell culture medium	0.20 ± 0.03	<0.0001
Cells	0.41 ± 0.03	0.003 ± 0.003
Cells + NPs (0 h)	1.44 ± 0.06	19.7 ± 1.5
Cells + NPs (1 day)	1.70 ± 0.11	22.1 ± 1.7
Cells + NPs (7 days)	1.54 ± 0.09	20.3 ± 1.9
Average	1.56 ± 0.11	20.7 ± 1.0
Normalized	1.14 (Fe_norm._)	20.7 (Pt_norm._)
Sum (Fe + Pt)_norm._	21.8 μgmL^−1^	
Initial suspension	100 μg mL^−1^	
Uptake (%)	21.8%	

The concentration of Fe in the samples was found to be 1.44, 1.70 and 1.54 μg mL^−1^ immediately after the incubation, 24 hours and 1 week after incubation, respectively. On average, the Fe concentration in the cells was found to be 1.56 μg Fe per mL. Moreover, in the case of the Fe concentration, naturally occurring Fe in the cells (0.41 ± 0.03 μg mL^−1^) has to be subtracted to obtain the Fe concentration only due to the presence of the Fe–Pt NPs. The normalized values for Fe are shown in Table 2 (labelled as Fe_norm._). The concentration of Pt in the samples was found to be 19.7, 22.1 and 20.3 μg mL^−1^ immediately after incubation, 24 hours and 1 week after incubation, respectively. On average, the cellular uptake was found to be 20.7 μg Pt per mL. Because the concentrations of Fe and Pt are not decreasing with time this indicates that the NPs remain internalized and are not excreted by the cells. The total amount of Fe and Pt in the sample incubated for 12 h with Fe–Pt NPs was found to be 21.8 μg mL^−1^. By dividing this value by the initial concentration of the NP suspension that was used in the experiment (100 μg mL^−1^), the cellular uptake was calculated to be 21.8%. In order to gain a better insight into the cellular uptake, the concentrations of Fe and Pt are expressed per one cell. Therefore, the Fe and Pt concentrations were divided by the total mass of the cells in the sample (number of cells in the sample × mass of one cell). Using this approach, the concentrations of 0.33 pg Fe per cell and 6.31 pg Pt per cell were calculated. The high cellular uptake determined using the ICP-MS analysis is in a good agreement with the data obtained with the electron microscopy, where the images showed a significant amount of NPs internalized by the cells.

#### 3.3.3 *In vitro* NMR relaxivities

The *T*_1_ and *T*_2_ relaxation times for the cells that were incubated with different concentrations of HCA-Fe–Pt NPs (0, 10, 50 and 100 μg mL^−1^ for 12 h) were measured and the results are listed in Table 3. The *T*_1_(0) and *T*_2_(0) values represent the 0 mM concentration, *i.e.*, control cells that were not incubated with NPs. In order to assess the decrease of the *T*_1_ or *T*_2_ value due to the presence of the hybrid NPs, the ratios *T*_1_/*T*_1_(0) and *T*_2_/*T*_2_(0) between each sample and the control sample were calculated. From the calculations in Table 3 it is clear that the incubation of the cells with Fe–Pt NPs having different concentrations (10, 50 and 100 μg mL^−1^) the *T*_1_ relaxation time decreased to 98, 72 and 71% of the initial *T*_1_(0) value due to the NPs' uptake. Furthermore, in the case of the *T*_2_ relaxation-time measurements it can be seen that the *T*_2_ values are decreased significantly to 30, 15 and 17% of the initial *T*_2_(0) value for the concentrations of Fe–Pt NPs of 10, 50 and 100 μg mL^−1^, respectively. Due to the superparamagnetic nature of the NPs, Fe–Pt NPs have a more significant effect on the shortening of *T*_2_ than the *T*_1_ relaxation time. The same behaviour was observed in *ex vitro* experiments measuring aqueous suspensions of Fe–Pt NPs.^6^ It seems that at higher incubation concentrations (50 and 100 μg mL^−1^) the saturation is achieved and the *T*_2_ values stabilized around 16% of the initial *T*_2_(0). It can be seen that the exposure of the cells to the lowest used concentration of NPs (10 μg mL^−1^) is enough to decrease the *T*_2_ value by 70%. In terms of the MRI imaging this means a large improvement in the contrast, even for low NP concentrations and easier visualization of the tissues containing NPs, proving that HCA-Fe–Pt NPs have great potential to be used as MRI contrast agents.

**Table 3:** List of *T*_1_ and *T*_2_ relaxation times obtained in *vitro* experiments for the cells that were incubated with different concentrations of HCA-Fe–Pt NPs (0, 10, 50 and 100 μg mL^−1^) with the corresponding calculated *T*_1_/*T*_1_(0) and *T*_2_/*T*_2_(0) ratios. For comparison, the values for pure water (*T*(0)-aq) and the aqueous suspension of Fe–Pt NPs (*T*(Fe–Pt)-aq) are added

Samples	Fe conc. (μg mL)	*T*_1_ (ms)	Ratio *T*_1_/*T*_1_(0)	*T*_2_ (ms)	Ratio *T*_2_/*T*_2_(0)
*T*(0)-cells	0	2340 ± 40	1	210 ± 10	1
*T*(10)-cells	N/A	2300 ± 40	0.98	63 ± 3	0.30
*T*(50)-cells	N/A	1690 ± 30	0.72	32 ± 2	0.15
*T*(100)-cells	1.14	1660 ± 30	0.71	36 ± 2	0.17
*T*(0)-aq	0	3089 ± 42	1	2077 ± 2	1
*T*(Fe–Pt)-aq	1.40	2206 ± 23	0.71	364 ± 2	0.18

Up to now, only one report could be found where *in vitro* MRI experiments were performed using Fe–Pt NPs.^14^ More precisely, HeLa cells were incubated with tetraethylene glycol/oleic acid-coated 4 nm-large Fe–Pt nanoparticles and the *r*_2_ was calculated to be 68.7 mM^−1^ s^−1^, which is lower than in an aqueous environment (*r*_2_ = 122 mM^−1^ s^−1^). Unfortunately, no explanation for this drop in the *r*_2_ value was given. Shortening the relaxation time depends on several parameters, such as the NPs' size and concentration, water accessibility and the distribution of NPs within the micro-environment.^41^ In suspensions the NPs are free to move and have easier access to the water protons. While in cells the NPs are usually trapped in the intracellular compartments and, therefore, their influence on the water protons could be weaker.^41^ Therefore, in order to assess the behaviour of the Fe–Pt NPs in a different environment, a comparison of the *T*_1_ and *T*_2_ relaxation times obtained for the Fe–Pt NPs in the water suspension and the Fe–Pt NPs internalized in the cells was performed. Iron, as the magnetic atom in the NPs, is responsible for the generation of the magnetic field, which influences the *T*_2_ relaxation time of the water protons; therefore, samples with the same Fe content were compared. For the cells incubated with the Fe–Pt suspension having a concentration of 100 μg mL^−1^, the Fe content in the cells was determined to be 1.14 μg mL^−1^ (Table 2). For comparison purposes, the *T*_1_ and *T*_2_ relaxation times for the aqueous suspension with the same Fe content were taken from our previous study^6^ and are listed in Table 3, where *T*(0)-aq stands for the pure water and *T*(Fe–Pt)-aq for the Fe–Pt suspension with an iron concentration of 1.40 μg mL^−1^. Calculations revealed that the *T*_1_/*T*_1_(0) and *T*_2_/*T*_2_(0) ratios for both samples, *i.e.*, the cell pellet and the aqueous suspension, having similar Fe contents, are comparable. This indicates that the Fe–Pt NPs in the cellular environment have a similar effect on the shortening of the *T*_1_ and *T*_2_ relaxation times as in the suspension. However, to determine the effect of the environment more precisely (water suspension *vs.* intracellular position) more detailed experiments with a larger population of the samples over a wider concentration range have to be performed. Relaxivity coefficients (*r*_2_ or *r*_1_, mM^−1^ s^−1^) are important parameters that define the ability to accelerate the relaxation rates (*R*_2_ or *R*_1_, s^−1^). In our previous study,^6^ relaxivity values for Fe–Pt clusters in an aqueous suspension were calculated to be *r*_1_ = 6.7 ± 0.7 mM^−1^ s^−1^ and *r*_2_ = 87 ± 4 mM^−1^ s^−1^ with an *r*_2_/*r*_1_ ratio 12.9, which put Fe–Pt nanoparticles high on the list of the most promising materials for use in MRI. We are aware that our *r*_2_ values for the Fe–Pt nanoparticles are, however, still inferior to iron oxides clusters, mainly due to their higher *m*_s_.^42–45^ But, despite their good magnetic properties, the Fe-oxide nanoparticles were found to have a limited *trans*-endothelia passage and tissue penetration, since they suffer from rapid clearance by phagocytic cells,^46,47^ which could be limiting in terms of their biomedical implementations. The MRI performance of the Fe–Pt NPs can be further improved with a careful preparation of NPs with the maximum *m*_s_ that can be achieved for this material in the superparamagnetic range. An important step towards the realization of this goal was achieved in the scope of this study, where the guidelines for the optimal NP size and morphology were given. Additionally, the surface layer can be optimized in the future in order to increase the *r*_2_. However, the biocompatibility of the selected ligand has to be taken into account to ensure the NPs' safety as well. Therefore, the development of new, biocompatible ligands, such as HCA, is of vital importance for the successful implementation of nanoparticulate systems to be used as safe and efficient MRI contrast agents.

## 4. Conclusions

Separated Fe–Pt NPs and Fe–Pt clusters were synthesized and the magnetic interactions were studied to obtain the optimal size and morphology that would lead to the highest possible *m*_s_ in the superparamagnetic regime. This was obtained by combining the NPs just below the *D*_SPL_, into clusters as a consequence of the exchange interactions acting between the individual Fe–Pt particles. Excellent stability of Fe–Pt NPs was achieved with a successful ligand exchange using a novel HCA ligand, the binding of which to the Fe–Pt NPs was proved *via* a FTIR study. The *in vitro* study made on HCA-functionalized Fe–Pt clusters revealed that the as-prepared NPs are not cytotoxic to the urothelial RT4 cell line, even at the highest used concentration (550 μg mL^−1^ of Fe–Pt NPs) that surpasses the concentrations used in the literature so far. Furthermore, no delayed cytotoxic effect was observed even 1 week after the cells' exposure to the NPs. A TEM analysis revealed a time-dependent cellular uptake. After 1 h of the incubation only a few NPs can be found on the cell membrane or internalized, while after 12 h the amount of internalized NPs is significantly higher and clearly visible intracellularly in the endosomal compartments. Despite the presence of the NPs, the cell organelles were found to be well preserved and of normal morphology, which further indicates the biocompatibility of HCA-Fe–Pt NPs. Moreover, an ICP-MS analysis was used to quantitatively assess the amount of NPs taken up. The results were in good agreement with the TEM observations. Finally, *in vitro* MRI measurements have shown that the exposure of the cells to the lowest used concentration of the HCA-Fe–Pt NPs (10 μg mL^−1^) is already enough to decrease the *T*_2_ value by 70%. Thus proving that HCA-Fe–Pt NPs have great potential for use as efficient and safe MRI contrast agents.

## Conflicts of interest

There are no conflicts to declare.

## Supplementary Material

RA-008-C8RA00047F-s001
