# Supplementary material for: Magnetic interactions and in vitro study of biocompatible hydrocaffeic acid-stabilized Fe–Pt clusters as MRI contrast agents
Source: RSC Adv. 2018 Apr 19;8(26):14694–704. doi: 10.1039/c8ra00047f (PMC9080024; doi:10.1039/c8ra00047f)
Supplement: RA-008-C8RA00047F-s001 [file RA-008-C8RA00047F-s001.pdf]

Electronic Supplementary Material (ESI) for RSC Advances.
This journal is © The Royal Society of Chemistry 2018

Electronic Supplementary Information

Magnetic interactions and *In Vitro* Study of Biocompatible Hydrocaffeic Acid-stabilized Fe-Pt Clusters as MRI Contrast Agents

N. Kostevšek,[a*] S. Hudoklin,[b] M.E. Kreft, [b] I. Serša,[c] A. Sepe,[c] Z. Jagličić,[d] J. Vidmar,[e] J. Ščančar,[e] S. Šturm,[a,f] S. Kobe[a,f] and K. Žužek Rožman[a,f]

a. *Department for Nanostructured Materials, Jožef Stefan Institute, Jamova 39, Ljubljana, Slovenia*
b. *Institute of Cell Biology, Faculty of Medicine, University of Ljubljana, Vrazov trg 2, Ljubljana, Slovenia*
c. *Department for Condensed Matter Physics, Jožef Stefan Institute, Jamova 39, Ljubljana, Slovenia*
d. *Institute of Mathematics, Physics and Mechanics & Faculty of Engineering and Geodesy, University of Ljubljana, Jadranska 19, 1000 Ljubljana, Slovenia*
e. *Department for environmental sciences, Jožef Stefan Institute, Jamova 39, Ljubljana, Slovenia*
f. *Jožef Stefan International Postgraduate School, Jamova 39, Ljubljana, Slovenia*

* Address correspondence to Nina Kostevšek: e-mail: nina.kostevsek@ijs.si

Superparamagnetic size limit for *fcc* Fe-Pt nanoparticles

Because in the literature no information about the superparamagnetic size limit (D_{SPL}) for *fcc* Fe-Pt can be found, we used experimentally determined blocking temperatures (T_b) from the temperature-dependent field-cooled and zero-field-cooled (FC/ZFC) magnetic susceptibility curves and effective anisotropy constant (K_{eff}) values from the literature for spherical *fcc* Fe-Pt NPs with different sizes to calculate D_{SPL}. When T_b and the size of the NPs are known, then the effective anisotropy constant K_{eff} for the non-interacting monodispersed NPs can be calculated using the following equation:

$$K_{eff} = \frac{25\, k_b\, T_b}{V}, \qquad \text{(S-1)}$$

where k_B is the Boltzmann constant, T_b is the blocking temperature and V is the volume of the particle. Furthermore, **Eq. (S-2)** describing the Néel relaxation time can be used for the estimation of the SPL.

$$\tau_N = \tau_0 \exp\left(\frac{K_{eff}V}{k_b T}\right), \qquad \text{(S-2)}$$

The superparamagnetic behaviour occurs when $k_b\, T >> K_{eff}V$ is valid and **Eq. (S-3)** can be rearranged into:

$$\left(\frac{K_{eff}V}{k_bT}\right) \geq \ln\left(\frac{\tau_m}{\tau_0}\right) \tag{S-3}$$

For the materials with high K_{eff}, such as *fct* Fe-Pt, *fct* CoPt, etc., in order to maintain the thermal stability of single-domain NPs, which is important, for example, in information storage [1], this ratio should be larger than 60:

$$\left(\frac{K_{eff}V}{k_bT}\right) \geq 60 \tag{S-4}$$

The diameter of a NP at the superparamagnetic size limit (D_{SPL}) can be calculated from the following equation:

$$D_{SPL} = \sqrt[3]{\frac{60\, k_bT_b}{K_{eff}}} \tag{S-5}$$

Because the value of K_{eff} is shape dependent [2], to make a more solid comparison, only data for spherical *fcc* Fe-Pt NPs were used. The calculated D_{SPL} values listed in **Table S-1** are in the size range 8.8–12.5 nm. Deviation in the results can contribute to the errors coming from the determination of T_b. A broader size distribution means a broader peak in the ZFC curve and, consequently, a less accurate determination of T_b. Moreover, the position of T_b also depends on the measurement time, which brings an additional error in the calculation. However, from the obtained data we can conclude that the D_{SPL} for spherical *fcc* Fe-Pt NPs is approximately 10 nm; therefore, in order to keep the NPs in the superparamagnetic regime, this size should not be exceeded.

Table S-1: List of T_b and K_{eff} values from the literature for the spherical *fcc* Fe-Pt NPs with different sizes that were used in the calculation of D_{SPL}.

**NPs size**	**T_b (K)**	**K_{eff} (J/m^3)**	**D_{SPL} (nm)**	**Ref.**
3 nm	15	$3.7 \cdot 10^5$	8.8	[3]
3 nm	15	$2.0 \cdot 10^5$	9.2	[4],[5]
4 nm	25	$2.6 \cdot 10^5$	9.9	[6]
4.6 nm	53	$3.6 \cdot 10^5$	8.8	[7]
5 nm	30	$1.6 \cdot 10^5$	11.2	[8]
5 nm	26	$1.4 \cdot 10^5$	11.4	[2]
8 nm	70	$9.0 \cdot 10^4$	11.2	[8]
9 nm	140	$1.3 \cdot 10^5$	12.5	[9], [10]

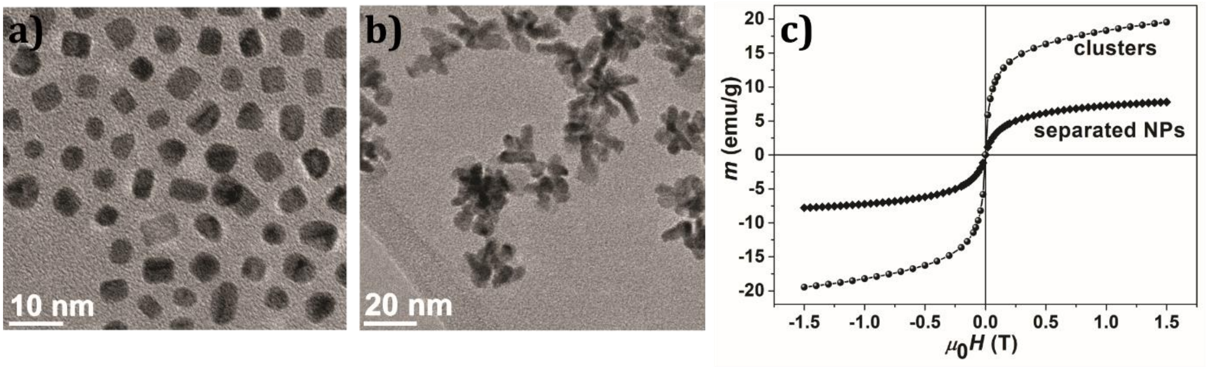

Figure S-1: TEM images of a) separated Fe-Pt NPs and b) Fe-Pt clusters and c) corresponding magnetic measurements at 300 K for both samples.

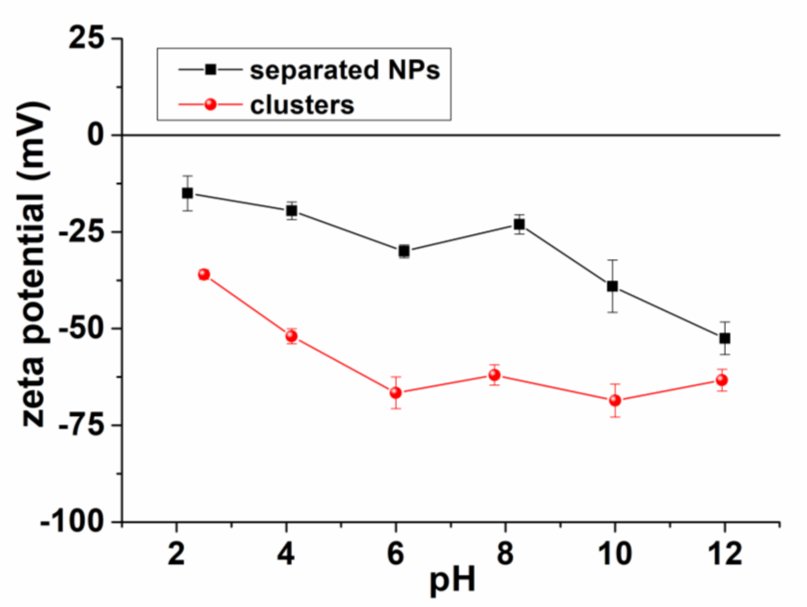

Figure S-2: Zeta-potential measurements of an aqueous suspensions of the separated Fe-Pt NPs and Fe-Pt clusters after ligand exchange with HCA.

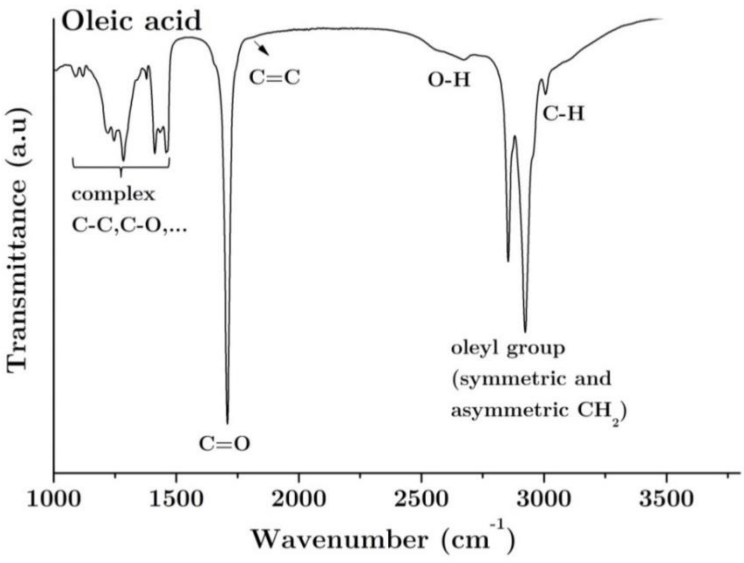

Figure S-3: FTIR spectrum of pure oleic acid (OA).

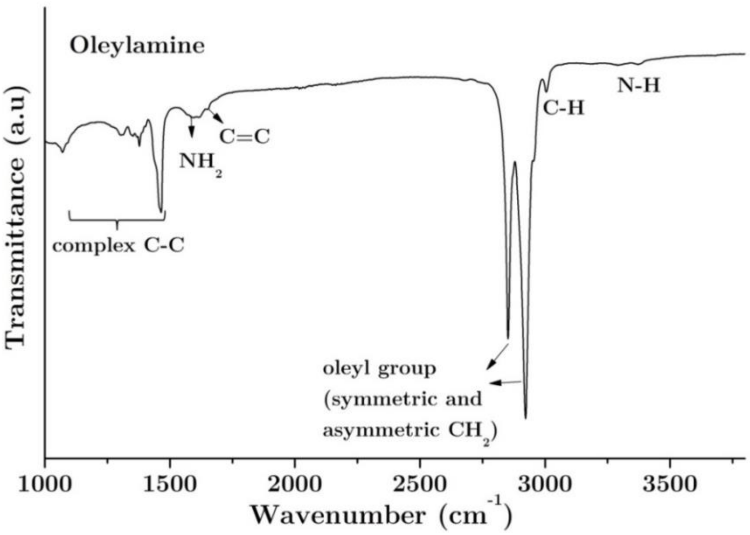

Figure S-4: FTIR spectrum of pure oleylamine (OLA).

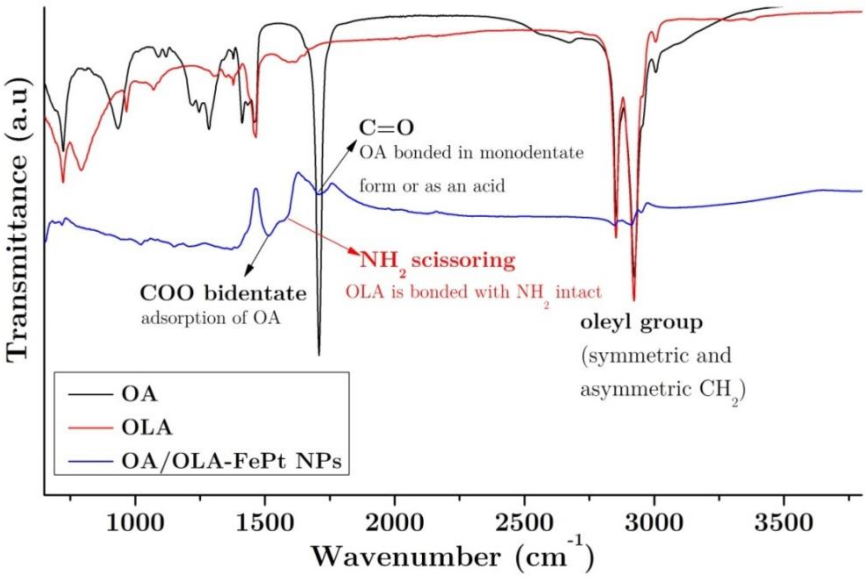

Figure S-5: FTIR spectra of pure oleic acid, pure oleylamine and oleic acid- and oleylamine-coated Fe-Pt NPs.

From the observation of ν(COO) and ν(C=O) vibrational modes it can be seen that OA bonds to the Fe-Pt NPs in both monodentate and bidentate forms (**Figure S-5**). More precisely, the peak at 1709 cm^{-1} corresponds to the ν(C=O) stretch mode and indicates that OA is bonded on the Fe-Pt NPs, either in monodentate form or as an acid. The peak at 1512 cm^{-1} corresponds to the ν(COO) mode and indicates the presence of the bidentate carboxylate bonding. The peak at 1590 cm^{-1} can be ascribed to the NH_2 scissoring, which suggests that OA is adsorbed, i.e., bonds to Fe-Pt NPs molecularly with the NH_2 group intact. This means that the OLA bonds to the Fe-Pt NPs through electron donation from the nitrogen atom of the NH_2 group [11]. Moreover, a broad peak with low intensity in the region 3000–3500 cm^{-1} can be attributed to the ν(NH) stretching mode, which confirms the previous argument about the NH_2 groups staying/being intact. Peaks at 2800–3000 cm^{-1} correspond to the symmetric and asymmetric CH_2 stretching modes of the oleyl group; however, they give no useful information about the bonding of OA or OLA on the surface of Fe-Pt NPs.

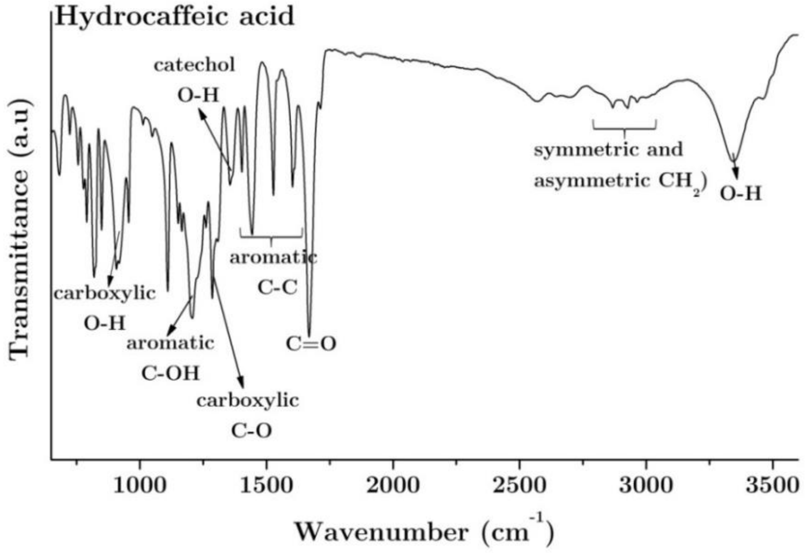

Figure S-6: FTIR spectrum of pure hydrocaffeic acid (HCA).

The FTIR spectrum of the pure HCA ligand is shown in **Figure S-5**. The broad peak centered at 3350 cm^{-1} can be attributed to the stretching vibration of the OH groups of HCA and the peak at 1360 cm^{-1} can be assigned to the bending vibrations of the catechol OH groups [11]. The peaks at 2800–3000 cm^{-1} correspond to the symmetric and asymmetric CH_2 stretching modes of the HCA molecule. The sharp peak at 1670 cm^{-1} corresponds to the *v*(C=O) stretch mode of the carboxylic group, which is found at a lower wavelength than expected for the monomeric acid (~1700 cm^{-1}) due to the intramolecular hydrogen bonding. The peaks from 1400–1600 cm^{-1} appear due to the C-C stretching in the aromatic ring. The peaks at 1290 cm^{-1} and 1200 cm^{-1} correspond to the *v*(C-O) stretch mode of the carboxylic group and *v*(C-OH) of the OH catechol group, respectively [13].

References

[1] N. a Frey and S. Sun, “Magnetic Nanoparticle for Information Storage Applications,” *Inorg. Nanoparticles Syntesis, Appl. Perspect.*, no. Richter, pp. 33–68, 2009.

[2] T. Şimşek and S. Özcan, “Effective magnetic anisotropy enhancement of Fe-Pt nanocrystals through shape control,” *J. Magn. Magn. Mater.*, vol. 351, pp. 47–51, 2014.

[3] C. B. Rong *et al.*, “Structural phase transition and ferromagnetism in monodisperse 3 nm Fe-Pt particles,” *J. Appl. Phys.*, vol. 102, no. 4, pp. 3–7, 2007.

[4] S. Maenosono and S. Saita, “Theoretical assessment of Fe-Pt nanoparticles as heating elements for magnetic hyperthermia,” *IEEE Trans. Magn.*, vol. 42, no. 6, pp. 1638–1642, 2006.

[5] M. S. Seehra *et al.*, “Size-dependent magnetic parameters of fcc Fe-Pt nanoparticles: applications to magnetic hyperthermia,” *J. Phys. D. Appl. Phys.*, vol. 43, no. 14, p. 145002, 2010.

[6] P. de la Presa, M. Multigner, M. P. Morales, T. Rueda, E. Fernandez-Pinel, and A. Hernando, “Synthesis and characterization of Fe-Pt/Au core-shell nanoparticles,” *J. Magn. Magn. Mater.*, vol. 316, no. 2 SPEC. ISS., 2007.

[7] B. Rellinghaus, S. Stappert, M. Acet, and E. F. Wassermann, “Magnetic properties of Fe-Pt nanoparticles,” *J. Magn.*

Magn. Mater., vol. 266, no. 1–2, pp. 142–154, 2003.

[8] V. Nandwana *et al.*, “Size and Shape Control of Monodisperse Fe-Pt Nanoparticles,” *J. Phys. Chem. C*, vol. 111, no. 11, pp. 4185–4189, 2007.

[9] Y. Tanaka, S. Saita, and S. Maenosono, “Influence of surface ligands on saturation magnetization of Fe-Pt nanoparticles,” *Appl. Phys. Lett.*, vol. 92, no. 9, pp. 47–50, 2008.

[10] S. Maenosono, T. Suzuki, and S. Saita, “Superparamagnetic Fe-Pt nanoparticles as excellent MRI contrast agents,” *J. Magn. Magn. Mater.*, vol. 320, no. 9, pp. 79–83, 2008.

[11] N. Shukla, C. Liu, P. M. Jones, and D. Weller, “FTIR study of surfactant bonding to Fe-Pt nanoparticles,” *J. Magn. Magn. Mater.*, vol. 266, no. 1–2, pp. 178–184, 2003.

[12] T. Rajh, L. X. Chen, K. Lukas, T. Liu, M. C. Thurnauer, and D. M. Tiede, “Surface Restructuring of Nanoparticles: An Efficient Route for Ligand−Metal Oxide Crosstalk,” *J. Phys. Chem. B*, vol. 106, no. 41, pp. 10543–10552, Oct. 2002.

[13] B. H. Stuart, “Spectral Analysis,” in *Infrared Spectroscopy: Fundamentals and Applications*, John Wiley & Sons, Ltd, 2005, pp. 45–70.
